# The Septic Theory of Dental Caries

**Published:** 1884-09

**Authors:** F. Searle

**Affiliations:** Springfield, Mass.


ARTICLE II.

### THE SEPTIC THEORY OF DENTAL CARIES.

BY F. SEARLE, D. D. S., SPRINGFIELD, MASS.

(Read before the Connecticut Valley Dental Society, June, 1884.)

In 1877 Arthur S. Underwood, M. R. C. S., L. D. S., England, and W. J. Milles, L. R. C. P., London, F. R. C. and C., undertook " to determine, as far as possible, to what extent germs are present in the dental tissue when in a state of disease, and to deduce conclusions as to the effect of their presence." After four years' patient investigation they published their first report of their progress in a paper read before the International Medical Congress, in which they say:

" We consider that caries is absolutely dependent upon the presence and prolification of organisms. This view we propose to call the ' septic theory.'"

In the examination of this theory, it will be necessary to go over a somewhat extended field of inquiry, and introduce some other theories for examination. Investigators into the phenomena of diseases are liable to seek an explanation in the insufficient evidence of their own discoveries, without taking into account what others have done or are doing in the study of questions bearing directly upon the subject which they are examining. Especially has this been the case with recent investigators of dental caries.

When Underwood and Milles in England were making their investigations in relation to organisms, they seemingly did not take into account the discoveries of Dr. Boedecker, of New York, in relation to the distribution of living matter in bone, dentine, enamel and cementum, or of the observations of Dr. Abbott respecting the evidence of inflammation in diseased fibrils. Dr. Abbott was equally uninformed of what Underwood and Milles were proving in regard to the presence and agency of germs in the fibrils. He saw the evidences of inflammation, but not, as I think, the true irritant. Underwood and Milles saw the irritant, but not the inflammatory appearances of the fibrils, or at least did not take them into account; consequently, they announced their septic theory, and Dr. Abbott his inflammation theory. The reviewer can gather and arrange all the evidences from all sources and thus form an unbroken chain of evidence, composed of demonstrated facts and legitimate deductions from those facts, which will explain more than any theory supported only by incomplete and one-sided observations.

I propose to gather up the important facts which have been reasonably well established, bearing upon the question of caries, separating them from mere opinions, and endeavor to find what they teach.

Our subject is intimately related to questions which have occupied the attention of chemists, for more than two hundred years. It is, in fact, one of the latest questions of a large and rapidly increasing number, growing out of those minute yeast granules which Leuwenhoeck discovered in 1680, and which Cagniard-Latour, and Schwann re-discovered in 1836–39, and which were the starting point of that long discussion between Pasteur and Liebig and their respective schools of the true theory of fermentation, which lasted from 1840 to 1870, and which the illustrious Pasteur has completely answered by a series of experiments which, in the language of Milles, are "classed among the most brilliant records in the history of science." "The true ferments are now known to be living organisms that produce fermentation as a function of vital activity." Unlike the soluble ferments, these living organisms increase at the expense of the substance fermented. The true fermentations are purely physiological processes, which are defined by Pasteur as the "direct consequence of the process of nutrition, assimilation and life when they are carried on without the aid of free oxygen, or as a result of life without air." (P. S. M., pp. 225.)

The discoveries of Schwann, Pasteur and others, in regard to the agency of the germs of micro-organisms, laid the foundation upon which Lister, 1865-67, established the germ theory oı contagious diseases, and the septic character of some diseases not so contagious, Lister, an eminent French surgeon, had previously exhausted all the resources of the old methods of dealing with septic diseases by efforts to exclude the oxygen of the air from wounds, etc., with unsatisfactory results. The revelations of Pasteur opened his eyes to the fact that his foundation principle was defective, and consequently his methods could not be suceessful. In 1867 he published the results of his antiseptic (and aseptic) methods which he had been testing for two years, with previously unheard-of success. This brought about the great revolution in general surgery which immediately followed, the principles of which have been generally accepted, and now direct the methods of surgery. Without the discoveries of Pasteur and Lister the germ theory of dental diseases would have long been deferred.

In 1873, Liebig and Rottenstein, published their work on Dental Caries and its Causes, which contains much of interest upon the existing theories, especially in relation to the germ theory, which they were the first to put into definite form. But the chemical theory held its ground in spite of their discoveries, because there was no Lister to give clinical demonstration of the correctness of their views. We hear but little more upon the subject in general allusions to the theory in partial approval, or total dissent, until 1881, when Underwood and Milles published their first paper.

The failure of the chemical theory by acids to explain satisfactorily all the conditions present in caries, led these gentlemen " to seek for the presence of organisms in carious dentine with results that far exceed their expectations."

Their first paper contains the following conclusions :

1. " We consider that caries is absolutely dependent upon the presence and prolification of organisms ; that these organisms attack first the organic material and, feeding upon it, create an acid which removes the lime-salts." . . .

2. " That suppuration of the pulp and its sequences, such as alveolar abscess, depend also upon the successful working of organisms."

In their second paper they say : " We are anxious that you should keep clearly before you four main issues upon which all the theory which we suggested to you in 1881 depends :

1. " That certain forms of micro-organisms, viz : Micrococci, oval bacteria and short bacilla, are invariably present in carious dentine."

2. " That these micro-organisms extend into the tissue as far as does the caries."

3. " That no agent can be made to produce a change resembling caries in the absence of such micro-organisms."

(The fourth I omit.)

They add : " We feel convinced that the process, to be effective, must be carried on in a living mouth, probably because that is the only situation in which the special germs are really active. Analogy seems to warrant this deduction; but, " all we claim for the germs is, that without them and their products the process could not go on at all," and " the dental pathology of the future must include them among the most powerful factors in the production of this disease."

To Underwood and Milles the world is indebted not only for the facts which they were the first to demonstrate, but also for the impulse which their labors gave to other men to make additions to their discoveries.

In the *New England Journal of Dentistry*, January, 1883, Prof. Mayr gives the results of a series of very careful experiments made in the line of analyzing decayed dentine. Of these experiments Underwood and Milles say: "Prof. Mayr has worked out the chemical aspect of the question with great care and exactness." That he is fully competent for a work of this kind, involving great care and labor, no candid man, who is at all acquainted with him personally, with his educational training in the best schools in Germany, his acquantance with medical and other sciences, and especially his ability in his chosen profession as a chemist, will for a moment question. I give the results of his experiments on the point bearing on the question of dental caries. First, he says: "Let us never confound two things; the structure of a tooth and the chemical nature of a tooth, and that the structure may be broken down and the chemical nature not changed. Result No. 1. No acids or chemical lime-salts are found in the innermost layer is almost (and as was seen in some cases completely) identical with that of healthy teeth, the structure is totally different. The soft, white, friable decay is no longer organized, though chemically only slightly differing from the tooth substance—one showing 2 per cent. lime-salts. The action at the innermost layer is not in accordance with what we know about the action of acids."

This paper of Prof. Mayr is full of suggestions and facts bearing directly upon the chemical aspect of the question of caries, and furnishing much evidence in favor of the septic theory.

Dr. W. D. Miller, of Berlin, within the last two years, has published many papers giving at length the processes of his experiments and observations relating to caries, which deserve notice. He then gives the description of the investigations which led him to several conclusions, of which I quote the following:

"The bacilla penetrate far into the dentine, even into the finest branches of the canaliculi. Micrococci penetrate furtherest" (*i. e.,* further than the minutest branches (?) ). "The fungi produce anatomical and pathological changes in the deeper layers, stop up the canaliculi and necessarily lead sooner or later to the death of the dentinal fibrils. The outer layers of dentine, thereby deprived of nourishment, die and fall a prey to putrefactive agents."

Dr. Miller thus fully confirms the observations of Underwood and Milles, of Lister, and Rottenstein, and every other careful observer, and the presence of organisms in the deeper portion of carious dentine may be considered as fully established.

Dr. Miller differs from Underwood and Milles in this: He claims that there is a zone of softened dentine between that occupied by the organisms and the healthy dentine, which is not infected. His opinion is based on microscopical observations, and this after having said that the bacilla penetrate even into the finest branches of the dentine (softened dentine), and the micrococci penetrate further still.

Underwood and Milles, on the other hand, prove that the germs are present up to the uttermost limit of the affected dentine, by inoculating a pure culture with matter taken from the deepest portions thereof—from the very border line of decay. In other words, they find them attacking the living *organic tissue* of dentine. The question of the effect of their presence here is now the one of paramount importance. The settlement of this very interesting question does not depend upon what organisms or acids may be doing at the more superficial points of the carious mass, nor whether the acids are organic or chemical, but what is going on at the line of battle where the amæba-like cells of the organisms and bioplasson are contending for dear life and for food to sustain that life.

Neither Underwood and Milles, nor Dr. Miller, are able to solve this question on the theory that organisms do their work by the acids which they produce. The former say: "The essential element wanting in our artificial experiments is present in the mouth." Dr. Miller says: "I am a believer in acids, a believer in micro-organisms, and a believer in an unknown cause; that is, I believe that there are agents active in the production of caries which are yet to be discovered.

Two questions here arise:

1. Is the tooth endowed with living matter capable of taking on inflammatory action?

2. Is there evidence to conclude that the results of inflammatory processes in dentine are analogous to those in bone and other tissues?

In answering the first question, I will refer you to the discoveries of Prof. Heitzmann in relation to the reticular structure of protoplasm, which connect every tissue and organ as it were into one large amæba, and to the researches of Dr. Bodecker, concerning "The Distribution of Living Matter in Human Dentine, Cement and Enamel," together with the evidence which your own experience furnished in your operations upon the teeth. If these, united, do not bring evidence to your minds sufficient to make you give an affirmative answer, ask your patients. Do not think, because these tissues belong to the microscopic world, that they cannot be injured and take on inflammation. All normal and abnormal life action, whether growth or decay, the building up or the taking down of molecules and tissues, belong to the microscopic world. If you discard the vital changes in one set of tissues, why not in all? Yes, there is the intensest vitality in the reticular matter of the tooth. To talk of its low vitality is to contradict the plainest evidence that can be brought to the human intellect.

In answer to the second question, if you admit the self-evident fact of its ability to live and grow and be healthy you must also admit its liability to disease.

This brings me to the consideration of the inflammation theory of Dr. Abbott.

It has been shown in the laboratory of Prof. Heitzmann that dentine has been dissolved and formation of abnormal tissue has taken its place. This has taken place by true physiological, though abnormal, vital processes. When Dr. Abbott was making his investigations in connection with Dr. Boedecker in regard to living matter in teeth, he, observed in the diseased fibrils of the tooth appearances characteristic of the presence of inflammation in other hard tissues, which he thus describes:

" First, irritation, which he thinks is produced by acids, followed by inflammation, swelling of the living matter, which effects the dislodgment of the lime-salts ; a melting down of the glue-giving basis-substance, and a bringing to view the embryonal elements of the enamel. These processes follow in greater intensity in the dentine. The lime-salts are not necessarily dissolved and taken away, but may, and I have no doubt, as Prof. Mayr's experiments show, do remain mixed with this disorganized tooth substance." On the hypothesis that acids produce the irritation of the living matter—the reticulum—at the border-line of decay, why should they not also decalcify the lime-salts at this line ? Prof. Mayr finds no trace of acids there, and Dr. Abbott accepts the conclusion that they are not there. How, then, can acids set up inflammation ? When the germ theory is accepted, the presence of acids at this point will be necessary to account for the agents of irritation. With vital organs feeding upon and multiplying within the pabulum contained the canaliculi, exhausting and injuring the reticulum, which is directly connected with the reticulum of the pulp and the whole system of tissues, the usual effects of irritation follow until the melting down of the formed material, the liberation of the lime-salts from its meshes and the death of the reticulum put an end to the vital reaction, and the mass is given up to the action of organisms and their products.

The facts already presented are sufficient to establish the septic character of the disease affecting the albuminous or "animal" portions of dentine where caries is present.

In another article I shall present some of the facts already discovered in regard to the action of pathogenic organisms on soft tissues, and also examine the chemical theory which teaches that caries is simply the decalcification of the dentine by acids.—*New England Journal of Dentistry.*

